# Egg and cholesterol intake, apoE4 phenotype and risk of venous thromboembolism: findings from a prospective cohort study

**DOI:** 10.1017/S0007114522000988

**Published:** 2023-01-28

**Authors:** Setor K. Kunutsor, Jari A. Laukkanen, Jyrki K. Virtanen

**Affiliations:** 1 National Institute for Health Research Bristol Biomedical Research Centre, University Hospitals Bristol and Weston NHS Foundation Trust and the University of Bristol, Bristol, UK; 2 Translational Health Sciences, Bristol Medical School, University of Bristol, Learning & Research Building (Level 1), Southmead Hospital, Bristol, UK; 3 Central Finland Health Care District, Department of Medicine, Jyväskylä, Finland; 4 Diabetes Research Centre, University of Leicester, Leicester General Hospital, Gwendolen Road, Leicester, LE5 4WP, UK; 5 Institute of Clinical Medicine, Department of Medicine, University of Eastern Finland, Kuopio, Finland; 6 Institute of Public Health and Clinical Nutrition, University of Eastern Finland, Kuopio, Finland

**Keywords:** Egg consumption, Dietary cholesterol, Venous thromboembolism, ApoE4, risk factor, Cohort study

## Abstract

The impact of egg consumption, a major source of dietary cholesterol, on the risk of atherosclerotic cardiovascular diseases (ASCVD) is controversial. Venous thromboembolism (VTE) is a CVD which shares common risk factors and mechanistic pathways with ASCVD. However, there is no data on the relationship between egg or cholesterol intake and VTE risk. Therefore, we evaluated the prospective associations of egg and cholesterol intakes with VTE risk and whether the apoE4 phenotype, which influences cholesterol metabolism, could modify the associations. Data involving 1852 men aged 42–61 years at baseline without a history of VTE or CHD in the population-based Kuopio Ischaemic Heart Disease Risk Factor Study were analysed. Dietary intakes were assessed with 4-d food records. Incident VTE events were identified by record linkage to hospital discharge registries. Hazard ratios (95 % CI) for incident VTE were estimated using Cox regression. During a median follow-up of 28·8 years, 132 VTE events occurred. Comparing the top (> 38 g/d) *v*. bottom (< 20 g/d) tertiles of egg consumption, the hazard ratio (95 % CI) for VTE was 0·99 (0·64, 1·53) in analysis adjusted for several established risk factors and other dietary factors. There was also no evidence of an association between cholesterol intake and VTE risk. Imputed results were consistent with the observed results. The apoE4 phenotype did not modify the associations. In middle-aged and older Finnish men, egg or cholesterol intakes were not associated with future VTE risk. Other large-scale prospective studies are needed to confirm or refute these findings.

Atherosclerotic cardiovascular disease (ASCVD) (arterial thrombotic disease), which includes CHD and cerebrovascular disease (ischemic stroke)^(1)^, is the major manifestation of CVD; CVD is the leading cause of morbidity and mortality globally^(1)^ and also associated with substantial costs to healthcare systems. Major risk factors for ASCVD include age, sex, blood cholesterol (lipids), blood pressure, diabetes and smoking status^(2)^. Venous thromboembolism (VTE) (comprising deep vein thrombosis and pulmonary embolism) is the third leading vascular disease after CHD and stroke^(3)^; it is also associated with significant morbidity and economic costs and is a preventable cause of death^(4,5)^. Emerging evidence suggests that ASCVD and VTE are closely related via shared risk factors such as age, obesity and cigarette smoking^(6,7)^ and pathophysiological pathways such as coagulation, platelet activation and dyslipidaemia^(8)^. The association between serum cholesterol and CHD risk has been documented as very strong^(9)^. It has been reported that dyslipidemia may not only be associated with arterial thrombotic disease, but with VTE as well^(8)^. Given that previous reports have shown that statins (lipid-lowering drugs) are associated with decreased risk of VTE^(10–13)^, there might be a potential role for lipids in the pathophysiology of VTE. In line with this plausibility, a number of observational studies have demonstrated associations between serum cholesterol parameters and VTE risk^(14,15)^.

Major advances have been made in the prevention of ASCVD through risk factor modification via physical activity, healthy dietary patters and lipid lowering. Though several factors explain a considerable proportion of VTE cases, the causes are still unknown in a substantial number of VTE cases^(16)^. Like ASCVD, VTE constitutes a major public health burden and there is a need to identify risk factors that could aid in the development of preventive strategies. There is evidence to suggest that the adoption of healthy lifestyles such as engaging in habitual physical activity and consuming a healthy diet could help prevent VTE^(17,18)^. Conversely, unhealthy lifestyles such as prolonged sedentary behaviours increase the risk of developing VTE^(19,20)^. Unlike ASCVD, the primary prevention of VTE through lifestyle modification has been largely ignored in guideline recommendations^(21)^.

Though dietary cholesterol does not make an appreciable contribution to serum cholesterol concentrations in most people^(22)^, associations between cholesterol intake and risk of ASCVD have been demonstrated in some studies^(23)^. The impact of egg consumption, a major source of dietary cholesterol (about 200 mg per medium-sized egg), on the risk of ASCVD and other cardiometabolic conditions such as type 2 diabetes is controversial. Meta-analyses of prospective cohort studies suggest little adverse cardiovascular effects with egg intake up to 1 egg per d^(24)^ but have rather observed an inverse association with risk of stroke^(25)^ and hypertension^(26)^. In studies conducted in the USA, egg intake has been associated with higher risk of type 2 diabetes, but such an association has not been observed in studies conducted in Europe or in Asia^(27)^. However, no study has previously assessed if a prospective association exists between egg or cholesterol intakes and future VTE risk. Given the nature of the overall existing evidence, we hypothesised that egg and cholesterol intakes will not be associated with the risk of VTE. In this context, our primary objective was to evaluate the prospective associations of egg and cholesterol intakes with the risk of VTE using a population-based prospective cohort of 1852 middle-aged Finnish men without VTE or CHD at baseline. Given that the influence of dietary cholesterol on serum LDL-cholesterol concentrations is more pronounced among individuals with apoE allele 4 (apoE4) in the general population^(28)^, a subsidiary analysis assessed if the apoE4 phenotype could modify the association between egg or cholesterol intakes and VTE risk.

## Materials and methods

### Study design and participants

Reporting of the study conforms to broad EQUATOR guidelines^(29)^ and was conducted according to STROBE (STrengthening the Reporting of OBservational studies in Epidemiology) guidelines for reporting observational studies in epidemiology (online Supplementary Material 1). The study protocol and design were approved by the Research Ethics Committee of the University of Kuopio. Each study participant provided written informed consent. All study procedures adhered to the Declaration of Helsinki. Study participants included in this analysis were part of the Kuopio Ischaemic Heart Disease Risk Factor Study (KIHD), a population-based prospective study designed to investigate risk factors for ASCVD and other related outcomes. Participants included in the KIHD comprised a representative sample of men living in the city of Kuopio and its surrounding rural communities in eastern Finland. Details of the study design and recruitment methods have been described in previous reports^(30–33)^. Briefly, participants were men aged 42, 48, 54, or 60 years during baseline examinations performed between March 1984 and December 1989. During recruitment, a total of 3433 men were potentially eligible and of these, 3235 were found to be eligible for inclusion into study. Of this number, 2682 volunteered to participate and 553 did not respond to the invitation or declined to give informed consent. From the analyses, we excluded (i) men with a history of CHD (*n* 677) as they are likely to have changed their diet as part of lifestyle modification or upon advice from a physician and (ii) those with missing data on dietary intakes and confounders (*n* 153). The current analysis included 1852 men with no previous history of VTE or CHD and complete information on egg consumption, dietary cholesterol intake, relevant covariates and first VTE events (online Supplementary Material 2).

### Measurement of covariates and outcome ascertainment

The collection of blood samples, physical measurements, assessment of lifestyle characteristics, medical history and dietary intakes, and measurement of blood biomarkers have been described in detail in previous reports^(34–36)^. For blood sample collection, participants fasted overnight and abstained from drinking alcohol for at least 3 d and from smoking for at least 12 h before blood samples were taken between 08.00 and 10.00. Smoking, alcohol consumption and medical history were assessed by self-administered questionnaires^(34)^. The apoE4 phenotype was determined from plasma using isoelectric focusing and immunoblotting techniques. Subjects who had the phenotype 3/4 or 4/4 were included in the apoE4 group. The consumption of foods was assessed with the use of a 4-d-guided food record, during three weekdays and one weekend day using household measures. A picture book of common foods and dishes was used to help in the estimation of portion sizes. Instructions were provided and completed food records were checked by a nutritionist together with the participant, to ensure accuracy. Nutrient intakes were estimated with the use of NUTRICA version 2.5 software (Social Insurance Institution, Finland), and these were energy-adjusted with the use of the residual method^(37)^. The egg consumption variable represented total egg consumption (g) per d and included the intake of eggs in mixed dishes and recipes. We included all first incident VTE events that occurred from study entry to 31 December 2018. All VTE events required positive imaging tests for their diagnoses and were identified by computer linkage to the National Hospital Discharge Registry data. Each event was validated by two physicians who were blinded to the exposures following detailed cross-checking of medical documents. The ICD 10 codes (I26, I80 and I82) were used to code and classify each VTE case.

### Statistical analysis

Baseline characteristics were presented as means and standard deviation or median (interquartile range) for continuous variables and percentages for categorical variables. To assess the cross-sectional associations of egg consumption with various risk markers, Pearson’s correlation coefficients were estimated using linear regression models adjusted for age. We also assessed univariable relationships between egg consumption and baseline characteristics using ANOVA (for continuous variables) and *χ*^2^ tests (for categorical variables). Hazard ratios with 95 % CI for incident VTE were estimated using Cox proportional hazard models after confirmation of no major departure from the proportionality of hazards assumptions using Schoenfeld residuals^(38)^. The adjustment for confounders were based on three models: (model 1) age and total energy intake; (model 2) model 1 plus systolic blood pressure, BMI, serum TAG, smoking status, alcohol consumption, leisure-time physical activity, socio-economic status, total energy intake, serum albumin, intake of fruits, berries and vegetables, intake of processed and unprocessed red meat, and history of cancer; and (model 3) a potential mediator-adjusted model comprising history of type 2 diabetes, serum total cholesterol, serum TAG and serum high-sensitivity C-reactive protein concentrations. The confounders selected for models 1–2 were based on their previously established roles as risk factors for VTE, evidence from previous research, previously published associations with VTE in the KIHD study^(39–41)^, or their potential as confounders based on known associations with VTE outcomes and observed associations with egg consumption using the available data^(42)^. Tests of interaction were used to formally assess if the risk of VTE associated with egg consumption and dietary cholesterol intake was modified by the apoE4 phenotype. We conducted multiple imputation by chained equations to handle potential selection bias originating from missingness. The imputation model included all model covariates as well as VTE outcome status. Given the computational time required, ten imputations were computed. Cox regression analyses were run across the ten imputed datasets, and the pooled estimates were reported. All statistical analyses were conducted using Stata version MP 16 (Stata Corp).

## Results

### Baseline characteristics

Table 1 shows baseline characteristics of study participants and cross-sectional correlates of egg consumption. The mean (standard deviation) age, egg consumption and dietary cholesterol intake of the 1852 men at baseline were 52 (5) years, 33 (26) g/d and 404 (107) mg/d, respectively. Egg consumption was weakly and inversely correlated with age, blood pressure and TAG. There were moderate to strong positive correlations with total energy intake and dietary cholesterol. At baseline, men with higher egg intake were less likely to smoke, have lower concentrations of serum TAG and high-sensitivity C-reactive protein, and had higher intakes of energy, dietary cholesterol and processed and unprocessed red meat (Table 2). Furthermore, men with lower egg intake were more likely to consume alcohol.

**Table 1.** Baseline participant characteristics and correlates of egg consumption (*n* 1852)

	Mean or %	sd	Pearson’s correlation r	95 % CI†	Percentage difference in values of egg consumption per 1 sd higher or compared with reference category of correlate‡	95 % CI
Egg consumption (g/d)	33	26	–		–	
Questionnaire/prevalent conditions						
Age at survey (years)	52	5	–0·06	–0·10, −0·01*	–1·44 %	–2·62, −0·27*
Alcohol consumption (g/week)						
Median	32·1		–0·02	–0·07, 0·03	–0·58 %	–1·8, 0·66
IQR	6·6–89·8					
Socio-economic status	7·99	4·22	0·04	–0·01, 0·08	0·94 %	–0·27, 2·14
History of type 2 diabetes						
No	1801	97·3	–		ref	
Yes	51	2·8	–		–4·30 %	–11·48, 2·88
Smoking status						
Other	1306	70·5	–		ref	
Current	546	29·5	–		–3·28 %	–5·86,–0·71*
History of cancer						
No	1823	98·4	–		ref	
Yes	29	1·6	–		–1·76 %	–11·23, 7·70
Physical measurements						
BMI (kg/m^2^)	26·7	3·5	–0·01	–0·05, 0·04	–0·20 %	–1·37, 0·98
SBP (mmHg)	134	17	–0·04	–0·08, 0·01	–0·93 %	–2·12, 0·25
DBP (mmHg)	89	11	–0·05	–0·09, −0·00*	–1·19 %	–2·37, −0·02*
Physical activity (kJ/d)						
Median	1199		–0·00	–0·05, 0·04	–0·10 %	–1·29, 1·08
IQR	642–1968					
Blood-based markers						
Albumin (g/l)	42·4	3·6	–0·01	–0·06, 0·03	–0·33 %	–1·51, 0·85
Total cholesterol (mmol/l)	5·86	1·03	–0·01	–0·05, 0·04	–0·16 %	–1·34, 1·02
HDL-C (mmol/l)	1·31	0·29	0·03	–0·01, 0·08	0·81 %	–0·37, 1·98
TAG (mmol/l)						
Median	1·08		–0·06	–0·10, −0·01*	–1·45 %	–2·63, −0·28*
IQR	0·78–1·52					
High-sensitivity CRP (mg/l)						
Median	1·19		–0·03	–0·08, 0·01	–0·89 %	–2·06, 0·29
IQR	0·66–2·20					
Dietary intakes						
Total energy intake (kJ/d)	9935	2575	0·24	0·20, 0·29***	6·38 %	5·23, 7·54***
Cholesterol (mg/d)	404	107	0·73	0·71, 0·75***	18·75 %	17·95, 19·56***
Processed and unprocessed red meat (g/d)	146	79	0·02	–0·02, 0·07	0·56 %	–0·65, 1·77
Fruits, berries and vegetables (g/d)	258	157	0·04	–0·01, 0·09	1·03 %	–0·14, 2·21

IQR, interquartile range; SBP, systolic blood pressure; DBP, diastolic blood pressure; CRP, C-reactive protein.

† Pearson’s correlation coefficients between egg consumption and the row variables.

‡ Percentage change in values of egg consumption per 1 sd increase in the row variable (or for categorical variables, the percentage difference in mean values of egg consumption for the category *v.* the reference); asterisks indicate the level of statistical significance: *P < 0·05; ***P < 0·001.

**Table 2.** Baseline participant characteristics according to egg consumption (Mean values and standard deviations; numbers and percentage; median values and interquartile range)

	Egg consumption tertiles						
	T1 (20 g/d)		T2 (20–38 g/d)		T3 (> 38 g/d)		
	Mean or %	sd	Mean or %	sd	Mean or %	sd	*P*
Questionnaire/prevalent conditions							
Age at survey (years)	53	5	52	5	52	5	0·19
Alcohol consumption (g/week)							
Median	36·6		29·9		32·0		0·036
IQR	7·6–105·3		6·5–81·3		6·4–86·5		
Socio-economic status	8·05	4·24	7·74	4·30	8·18	4·09	0·18
History of type 2 diabetes							
No	622	96·3	605	97·9	574	97·6	0·17
Yes	24	3·7	13	2·1	14	2·4	
Smoking status							
Other	414	64·1	450	72·8	442	75·2	< 0·01
Current	232	35·9	168	27·2	146	24·8	
History of cancer							
No	636	98·5	608	98·4	579	98·5	0·99
Yes	10	1·5	10	1·6	9	1·5	
Physical measurements							
BMI (kg/m^2^)	26·8	3·6	26·7	3·4	26·7	3·3	0·86
SBP (mmHg)	135	17	135	17	133	16	0·084
DBP (mmHg)	90	11	89	11	88	10	0·015
Physical activity (kJ/d)							
Median	1151		1239		1220		0·17
IQR	631–1958		679–1940		606–2014		
Blood-based markers							
Albumin (g/l)	42·3	3·8	42·5	3·70	42·3	3·1	0·39
Total cholesterol (mmol/l)	5·89	1·07	5·81	1·04	5·88	0·99	0·35
HDL-C (mmol/l)	1·30	0·29	1·29	0·28	1·33	0·30	0·11
TAG (mmol/l)							
Median	1·15		1·07		1·03		0·016
IQR	0·80–1·59		0·80–1·53		0·75–1·41		
High-sensitivity CRP (mg/l)							
Median	1·24		1·16		1·15		0·018
IQR	0·67–1·59		0·65–2·16		0·64–2·07		
Dietary intakes							
Total energy intake (kJ/d)	9204	2503	9894	2432	10 782	2548	< 0·001
Cholesterol (mg/d)	343	73	385	73	492	112	< 0·001
Processed and unprocessed red meat (g/d)	143	82	145	77	149	76	0·35
Fruits, berries and vegetables (g/d)	245	167	259	148	270	153	0·022

T, tertile; IQR, interquartile range; SBP, systolic blood pressure; DBP, diastolic blood pressure; CRP, C-reactive protein.

### Associations of egg and cholesterol intake with venous thromboembolism risk

**Fig. 1.**
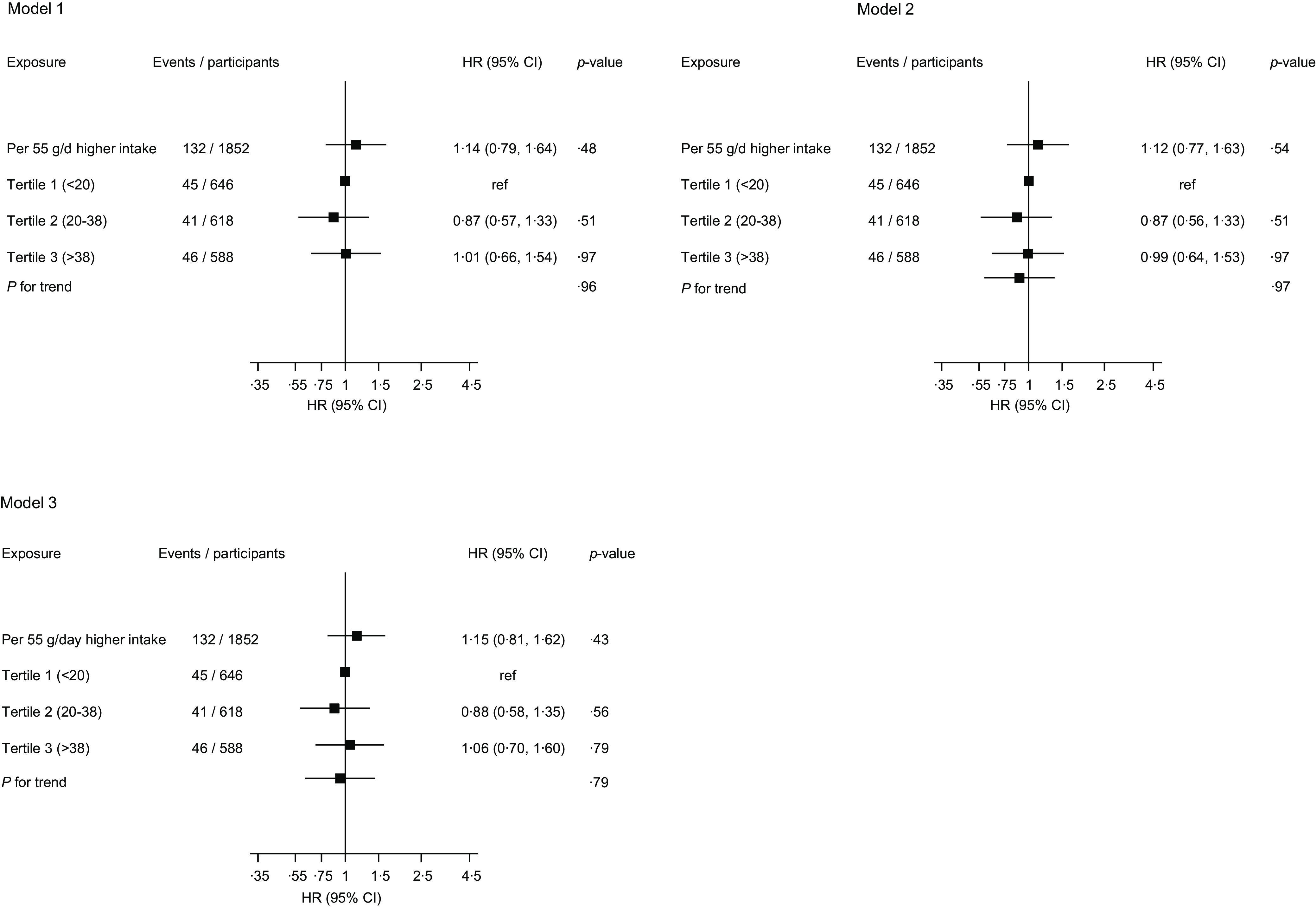
Association between egg consumption and risk of venous thromboembolism. HR, hazard ratio; ref, reference. Model 1: adjusted for age and total energy intake. Model 2: model 1 plus total energy intake, systolic blood pressure, BMI, serum TAG, smoking status, alcohol consumption, physical activity, socio-economic status, serum albumin, intake of fruits, berries and vegetables, intake of processed and unprocessed red meat, and history of cancer. Model 3: history of type 2 diabetes, serum total cholesterol, serum TAG and serum high-sensitivity C-reactive protein.

**Fig. 2.**
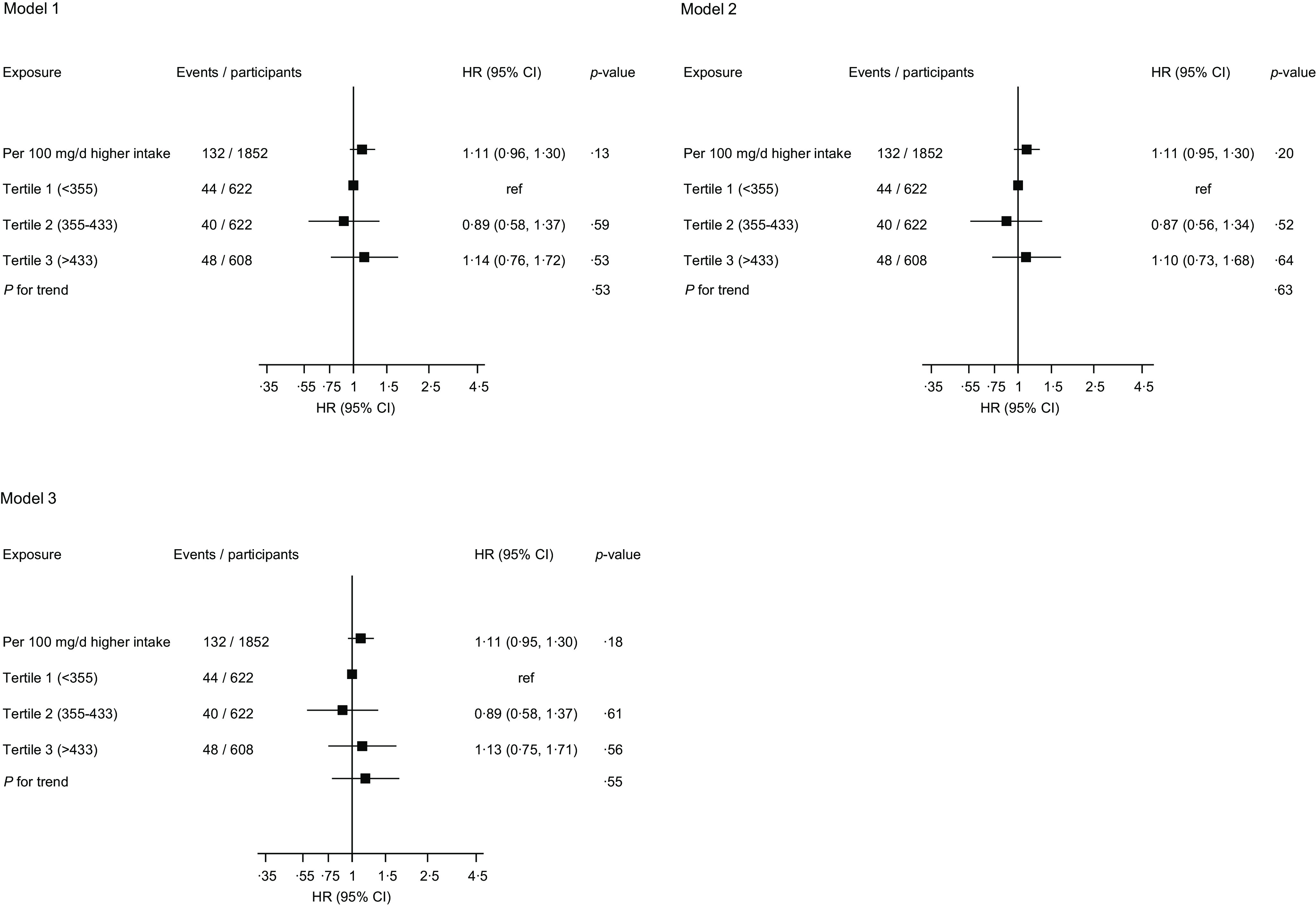
Association between dietary cholesterol intake and risk of venous thromboembolism. HR, hazard ratio; ref, reference. Model 1: adjusted for age and total energy intake. Model 2: model 1 plus total energy intake, systolic blood pressure, BMI, serum TAG, smoking status, alcohol consumption, physical activity, socio-economic status, serum albumin, intake of fruits, berries and vegetables, intake of processed and unprocessed red meat, and history of cancer. Model 3: history of type 2 diabetes, serum total cholesterol, serum TAG and serum high-sensitivity C-reactive protein

A total of 132 VTE events were recorded during a median (interquartile range) follow-up of 28·8 (19·6, 31·2) years. In analysis adjusted for age and total energy intake, the hazard ratio (95 % CI) for VTE comparing the top *v*. bottom tertiles of egg consumption was 1·01 (0·66, 1·54), which remained non-significant 0·99 (0·64, 1·53) on further adjustment for systolic blood pressure, BMI, TAG, smoking status, alcohol consumption, physical activity, socio-economic status, serum albumin, intake of fruits, berries and vegetables, intake of processed and unprocessed red meat, and history of cancer (Fig. 1). There was no evidence of an association in the model that adjusted for potential mediators (Fig. 1). The results were similar for the association between dietary cholesterol intake and VTE risk (Fig. 2). The null associations persisted when both exposures were modelled as continuous variables (Fig. 1 and 2). Data were imputed for 2005 participants, and the imputed results were consistent with those obtained using observed values (online Supplementary Materials 3–4).

Following exclusions of men with pre-existing CHD and missing data on dietary intakes and relevant confounders, there were 988 men with available data on apoE phenotype: 335 were carriers of the apoE4 phenotype (n of VTE events = 17) and 653 were non-carriers (n of VTE events = 54). For each 55 g/d (1 egg) higher egg intake, the hazard ratio (95 % CI) for VTE (based on model 2) were 1·11 (0·33, 3·72) and 1·17 (0·63, 2·16) for apoE4 carriers and non-carriers, respectively (*P*-value for interaction = 0·94). For each 100 mg/d higher cholesterol intake, the hazard ratio (95 % CI) for VTE (based on model 2) were 1·10 (0·69, 1·76) and 1·17 (0·91, 1·49) for apoE4 carriers and non-carriers, respectively (*P*-value for interaction = 0·83).

## Discussion

In this general population-based cohort of middle-aged and older Finnish men without a history of VTE or CHD, there were generally weak correlations of egg consumption with several VTE risk markers. In analysis adjusted for several established and emerging risk factors, egg consumption or dietary cholesterol intake was not associated with VTE risk, not even in participants with the apoE4 phenotype. The imputed results were similar to the observed results.

Given that this is the first reported evaluation of the prospective associations of egg and cholesterol intakes with VTE risk, the current findings cannot be discussed in comparison with previous studies. Other large-scale studies will be needed to refute or confirm these findings. However, given our previous findings in the KIHD cohort of no evidence of associations between egg or cholesterol intakes and carotid atherosclerosis or risk of CHD^(43)^ or stroke^(36)^, not even among the carriers of the apoE4, and an inverse association with type 2 diabetes^(44)^, the null findings with VTE risk are not that unexpected. There is a possibility that the absence of evidence of an association may represent the true relationship between egg consumption, a major source of dietary cholesterol, and VTE risk. Serum cholesterol is well established to be strongly associated with the risk of ASCVD. For several years, dietary cholesterol was implicated to increase serum cholesterol levels, leading to an increased risk of ASCVD^(45)^. As a result, major guideline bodies such as the American Heart Association made recommendations of limiting dietary cholesterol intake to 300 mg/d in healthy individuals and restricting egg consumption to not more than three whole eggs per week^(46)^. However, there is convincing evidence that dietary cholesterol only makes modest contributions to serum cholesterol concentrations in general populations^(22)^. There is also evidence suggesting that increased dietary cholesterol intake decreases the synthesis of endogenous de novo cholesterol, to maintain cholesterol homoeostasis^(47)^. An extensive review of observational and experimental research also does not provide conclusive evidence that dietary cholesterol is involved in the development of ASCVD^(45)^. This has led to the removal of recommendations restricting dietary cholesterol intake to 300 mg/d in the 2015–2020 Dietary Guidelines for Americans^(48)^. It has been reported that the observed association between dietary cholesterol and ASCVD could be driven by SFA which are high in foods containing dietary cholesterol^(45)^. SFA, especially when replacing PUFA in diet, are well known to increase the levels of LDL-cholesterol, which are associated with an increased risk of ASCVD^(49)^.

Though there is a wealth of evidence supporting a close relationship between ASCVD and VTE, definite conclusions have not been drawn as evidence on their shared risk factors and mechanisms has not been consistent. With regard to shared risk factors, some studies have demonstrated associations between traditional ASCVD risk factors and VTE risk^(7,50)^, whereas others have not^(51,52)^. While some studies have reported that ASCVD is an underlying condition and precedes the development of VTE^(53)^, other studies have shown that ASCVD does not precede VTE development^(54,55)^ or VTE rather precedes ASCVD^(56)^. Historically, ASCVD and VTE have been viewed as distinct pathophysiological entities, as a result of the obvious anatomical differences and their distinct clinical presentations^(57)^. Consistent with this is the inability of our study to demonstrate an association in apoE4 carriers, given that the subjects with the apoE4 phenotype have more pronounced elevations in LDL-cholesterol levels due to dietary cholesterol intake^(28)^ and higher risk of ASCVD^(58,59)^. Other potential reasons for the null results include the low event rate and underestimation of the true strength of the association due to regression dilution bias, given the use of only one dietary assessment at the baseline and the long follow-up duration. Due to the low incidence rates of VTE and hence few events in the first few years of follow-up, we were unable to conduct sufficiently powered targeted analyses to ascertain if the observed null associations could be due to potential regression dilution bias. However, our use of multiple imputation methods (based on 2005 participants) showed that the results of our complete-case analyses were not biased. Taking the overall evidence together and contrary to what was previously thought, it appears that dietary cholesterol intakes (including egg consumption) may not be associated with an increased risk of venous and arterial thromboembolic conditions, which are associated with substantial morbidity, premature mortality and high economic costs. Eggs are nutrient-dense food items, rich in several micronutrients including vitamins and minerals, and contain high-quality protein with minimal SFA (1·56 g/egg)^(45)^. Hence, it is appropriate to include moderate egg consumption as part of a healthy eating pattern.

We have conducted the first evaluation of the temporal relationships of egg and cholesterol intakes with VTE risk. Other strengths include the population-based prospective cohort design and the ability to assess if the apoE4 phenotype modified the associations. This was especially relevant given that the apoE4 phenotype is very common among the Finnish population; it has been reported that about one-third of the Finnish population possess ≥ 1 E4 allele^(60)^. There were limitations which deserve consideration and include as follows: (i) dietary intake was only assessed at baseline, which did not consider the possible dietary changes during the long follow-up; (ii) possible random errors in food recording that could attenuate the true associations; (iii) lack of specific data on nutrient intake from a specific method of preparation; however, all intake of nutrients including egg consumption represented total consumption and these included intake in mixed dishes and recipes; (iv) inability to generalise the findings to women and other populations; (v) inability to adjust for examination year as a number of participants (*n* 395) did not have the specific year of examination assigned to them; however, it is unlikely this would have impacted on our results given that the examination years spanned a relatively short period from 1984 to 1989; (vi) the relatively low event rate; (vii) lack of specific data on deep vein thrombosis and pulmonary embolism; and (viii) potential for biases due to the observational design. Given the limitations and being the first evaluation of its kind, caution is needed in interpreting the findings; they should be regarded mainly as hypothesis generating.

In summary, egg or cholesterol intake was not associated with future VTE risk in middle-aged and older men. Furthermore, apoE4 phenotype did not modify the associations. Other large-scale prospective studies conducted in other populations are needed to confirm or refute these findings.

## Supplementary material

For supplementary material accompanying this paper visit https://doi.org/10.1017/S0007114522000988. click here to view supplementary material
