# Supplementary material for: Egg and cholesterol intake, apoE4 phenotype and risk of venous thromboembolism: findings from a prospective cohort study
Source: Br J Nutr. 2022 Apr 21;129(2):292–300. doi: 10.1017/S0007114522000988 (PMC9870718; doi:10.1017/S0007114522000988)
Supplement: Supplementary file 1 [file S0007114522000988sup001.docx]

**Supplementary Materials**

| **Supplementary Material 1** | STROBE 2007 Statement—Checklist of items that should be included in reports of cohort studies |
| --- | --- |
| **Supplementary Material 2** | Flow of study participants |
| **Supplementary Material 3** | Imputed results of the association between egg consumption and risk of venous thromboembolism |
| **Supplementary Material 4** | Imputed results of the association between dietary cholesterol intake and risk of venous thromboembolism |

**Supplementary Material 1.** STROBE 2007 Statement—Checklist of items that should be included in reports of cohort studies

| **Section/Topic** | Item # | Recommendation | Reported on page # |
| --- | --- | --- | --- |
| **Title and abstract** | 1 | (*a*) Indicate the study’s design with a commonly used term in the title or the abstract | Page 1 |
|  |  | (*b*) Provide in the abstract an informative and balanced summary of what was done and what was found | Page 2 |
| Introduction | | |  |
| Background/rationale | 2 | Explain the scientific background and rationale for the investigation being reported | Pages 3-4 |
| Objectives | 3 | State specific objectives, including any prespecified hypotheses | Page 4 |
| Methods | | |  |
| Study design | 4 | Present key elements of study design early in the paper | Methods |
| Setting | 5 | Describe the setting, locations, and relevant dates, including periods of recruitment, exposure, follow-up, and data collection | Methods |
| Participants | 6 | (*a*) Give the eligibility criteria, and the sources and methods of selection of participants. Describe methods of follow-up | Methods |
|  |  | (*b*) For matched studies, give matching criteria and number of exposed and unexposed | Not applicable |
| Variables | 7 | Clearly define all outcomes, exposures, predictors, potential confounders, and effect modifiers. Give diagnostic criteria, if applicable | Methods |
| Data sources/ measurement | 8* | For each variable of interest, give sources of data and details of methods of assessment (measurement). Describe comparability of assessment methods if there is more than one group | Methods |
| Bias | 9 | Describe any efforts to address potential sources of bias | Methods |
| Study size | 10 | Explain how the study size was arrived at | Methods |
| Quantitative variables | 11 | Explain how quantitative variables were handled in the analyses. If applicable, describe which groupings were chosen and why | Methods |
| Statistical methods | 12 | (*a*) Describe all statistical methods, including those used to control for confounding | Methods |
|  |  | (*b*) Describe any methods used to examine subgroups and interactions | Methods |
|  |  | (*c*) Explain how missing data were addressed | Not applicable |
|  |  | (*d*) If applicable, explain how loss to follow-up was addressed | Not applicable |
|  |  | (*e*) Describe any sensitivity analyses | Methods |
| Results | | |  |
| Participants | 13* | (a) Report numbers of individuals at each stage of study—eg numbers potentially eligible, examined for eligibility, confirmed eligible, included in the study, completing follow-up, and analysed | Methods |
|  |  | (b) Give reasons for non-participation at each stage | Methods |
|  |  | (c) Consider use of a flow diagram |  |
| Descriptive data | 14* | (a) Give characteristics of study participants (eg demographic, clinical, social) and information on exposures and potential confounders | Results; Table 1 |
|  |  | (b) Indicate number of participants with missing data for each variable of interest |  |
|  |  | (c) Summarise follow-up time (eg, average and total amount) | Results |
| Outcome data | 15* | Report numbers of outcome events or summary measures over time | Results |
| Main results | 16 | (*a*) Give unadjusted estimates and, if applicable, confounder-adjusted estimates and their precision (eg, 95% confidence interval). Make clear which confounders were adjusted for and why they were included | Results; Table 2; |
|  |  | (*b*) Report category boundaries when continuous variables were categorized | Results; Table 2 |
|  |  | (*c*) If relevant, consider translating estimates of relative risk into absolute risk for a meaningful time period |  |
| Other analyses | 17 | Report other analyses done—eg analyses of subgroups and interactions, and sensitivity analyses | Results; Supplementary Materials 3-4 |
| Discussion |  |  |  |
| Key results | 18 | Summarise key results with reference to study objectives | Discussion |
| **Limitations** |  |  |  |
| Interpretation | 20 | Give a cautious overall interpretation of results considering objectives, limitations, multiplicity of analyses, results from similar studies, and other relevant evidence | Discussion |
| Generalisability | 21 | Discuss the generalisability (external validity) of the study results | Discussion |
| Other information |  |  |  |
| Funding | 22 | Give the source of funding and the role of the funders for the present study and, if applicable, for the original study on which the present article is based | After Discussion |

**Supplementary Material 2.** Flow of study participants

CHD, coronary heart disease; VTE, venous thromboembolism

**Supplementary Material 3.** Imputed results of the association between egg consumption and risk of venous thromboembolism

| **Egg consumption (g/day)** | **Model 1** | | **Model 2** | | **Model 3** | |
| --- | --- | --- | --- | --- | --- | --- |
|  | HR (95% CI) | *p-*value | HR (95% CI) | *p*-value | HR (95% CI) | *p-*value |
| Per 55 g/day higher intake | 1.16 (0.80-1.66) | .43 | 1.15 (0.79-1.66) | .46 | 1.16 (0.82-1.63) | .40 |
| T1 (<20) | ref |  | ref |  | ref |  |
| T2 (20-38) | 0.80 (0.52-1.23) | .30 | 0.81 (0.52-1.25) | .34 | 0.81 (0.53-1.25) | .35 |
| T3 (>38) | 1.02 (0.68-1.54) | .91 | 1.01 (0.67-1.54) | .95 | 1.06 (0.71-1.58) | .78 |

CI, confidence interval; HR, hazard ratio; ref, reference; T, tertile

Model 1: Adjusted for age and total energy intake

Model 2: Model 1 plus total energy intake, systolic blood pressure, body mass index, serum triglycerides, smoking status, alcohol consumption, physical activity, socioeconomic status, serum albumin, intake of fruits, berries and vegetables, intake of processed and unprocessed red meat, and history of cancer

Model 3: History of type 2 diabetes, serum total cholesterol, serum triglycerides and serum high-sensitivity C-reactive protein

**Supplementary Material 4.** Imputed results of the association between dietary cholesterol intake and risk of venous thromboembolism

| **Dietary cholesterol intake (mg/day)** | **Model 1** | | **Model 2** | | **Model 3** | |
| --- | --- | --- | --- | --- | --- | --- |
|  | HR (95% CI) | *p-*value | HR (95% CI) | *p*-value | HR (95% CI) | *p-*value |
| Per 100 mg/day higher intake | 1.12 (0.96-1.30) | .15 | 1.11 (0.95-1.30) | .19 | 1.11 (0.95-1.30) | .18 |
| T1 (<355) | ref |  | ref |  | ref |  |
| T2 (355-433) | 1.05 (0.69-1.61) | .82 | 1.04 (0.68-1.61) | .85 | 1.04 (0.68-1.59) | .87 |
| T3 (>433) | 1.16 (0.76-1.77) | .48 | 1.13 (0.74-1.74) | .57 | 1.15 (0.75-1.71) | .53 |

CI, confidence interval; HR, hazard ratio; ref, reference; T, tertile

Model 1: Adjusted for age and total energy intake

Model 2: Model 1 plus total energy intake, systolic blood pressure, body mass index, serum triglycerides, smoking status, alcohol consumption, physical activity, socioeconomic status, serum albumin, intake of fruits, berries and vegetables, intake of processed and unprocessed red meat, and history of cancer

Model 3: History of type 2 diabetes, serum total cholesterol, serum triglycerides, and serum high-sensitivity C-reactive protein
